# Hexahydrocannabinol Pharmacology, Toxicology, and Analysis: The First Evidence for a Recent New Psychoactive Substance

**DOI:** 10.2174/1570159X21666230623104624

**Published:** 2023-09-25

**Authors:** Silvia Graziano, Maria Rosaria Varì, Simona Pichini, Francesco Paolo Busardò, Tommaso Cassano, Annagiulia Di Trana

**Affiliations:** 1 National Centre on Addiction and Doping, Istituto Superiore di Sanità, 00161, Rome, Italy;; 2 Department of Excellence-Biomedical Sciences and Public Health, Università Politecnica delle Marche, Ancona, Italy;; 3 Department of Medical and Surgical Sciences, University of Foggia, Via Luigi Pinto, c/o Policlinico “Riuniti” di Foggia, 71122, Foggia, Italy

**Keywords:** Hexahydrocannabinol, cannabinoids, phytocannabinoids, new psychoactive substances, synthetic cannabinoids

## Abstract

**Background:**

During the last two years, hexahydrocannabinol (HHC), the hydrogenated derivative of tetrahydrocannabinol has been freely sold by internet websites as a “legal” replacement to THC and cannabis in a range of highly attractive branded and unbranded products, some of which are sold as “legal highs”. Potentially, there could be a large demand for HHC products by individuals in Europe and internationally.

**Methods:**

Studies reporting HHC pharmacology, toxicology and analysis were identified from Pubmed and Scopus databases, and official international organizations’ websites were considered.

**Results:**

HHC showed the effects of the typical cannabinoid on the central nervous system, with lower potency than Δ^9^-THC. A few studies highlighted that **9(R)**-HHC is more potent than **9(S)**-HHC. This molecule showed an affinity for cannabinoid receptor CB1 both *in vitro* and *in vivo*, suggesting a possible therapeutic effect in several pathologies. However, the affinity for the CB1 receptor suggests a possible addiction potential, inducing the users to misuse it. Since actual intoxication cases have not yet been reported, the HHC harmful potential was not described, probably due to the lack of effective analytical methods to detect HHC in biological matrices. Conversely, different analytical assays were developed and validated to separate HHC epimers in natural and non-natural sources.

**Conclusion:**

Similarly to other NPS, the HHC represents a cheaper alternative to the controlled Δ**^9^**-THC. Its monitoring is a crucial challenge for toxicological and forensic purposes. To this concern, it is essential to further investigate HHC to support health providers in the identification of related intoxications.

## 1 INTRODUCTION

**Fig. (1):**
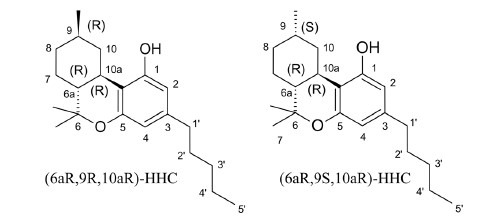
**9(R)-**HHC and **9(S)**-HHC molecular structures.

Hexahydrocannabinol (IUPAC name 6a,7,8,9,10,10a-hexahydro-6,6,9-trimethyl-3-pentyl-6*H*-dibenzo[b,d]pyran-1-ol, HHC) is a hydrogenated derivative of delta-9-tetrahydrocannabinol (Δ^9^-THC) and a phytocannabinoid naturally occurring in cannabis plant preparations in trace quantities [1-4]. The molecular scaffold of HHC (Fig. **1**) contains three stereogenic centers, with eight possible existing stereoisomers, whereas the optical configuration of carbons 6 and 10 was observed only as R, the chiral carbon 9 is naturally present in both configurations (R or S). As a result, two different epimers are known, (6aR,9R,10aR)-HHC (β-HHC) and (6aR,9S,10aR)-HHC (α-HHC) [1].

Due to the pharmacological activity of cannabinoids, HHC was initially considered a potential alternative to Δ^9^-THC, the principal constituent of *Cannabis sativa* [1]. The first HHC synthesis was reported by Adams *et al.* in 1940, who directly reduced natural delta-8-tetrahydrocannabinol (Δ^8^-THC) or Δ^9^-THC by catalytic hydrogenation [4, 5]. Then, several methods were explored to improve the stereoselectivity or the reaction efficiency to synthesize HHC from different sources [6]. Recently, the enantioselective total synthesis of HHC was achieved by online-catalyzed inverse-electron-demand Diels-Alder reaction [7]. Along with cannabinol (CBN), a trace of HHC may also occur in aged cannabis samples as a product of photochemical degradation of cannabidiol (CBD) or disproportionation of Δ^9^-THC [8]. Typically, cannabis-derived HHC is a mixture of **9(S)**- and **9(R)**-methyl stereoisomers. Although the pharmacological interest in HHC decayed rapidly after its synthesis in 1940, the structural studies on this molecule were particularly useful in elucidating important aspects of the endocannabinoid system [9]. Furthermore, the structural elucidation of HHC showed the importance of C9 substituent on cannabinoid scaffold and of the lone pairs of phenyl group hydroxyl oxygen orientation for the cannabimimetic activity of cannabinoids [10]. Recently, the HHC analogues have been investigated for pharmacological activity other than the usual cannabinoids, such as antitumoral activity [11].

Currently, HHC is not scheduled under the 1961 and 1971 United Nations drug conventions, and since the last two years, it has started to be freely sold mainly by internet websites as a “legal” replacement to Δ^9^-THC and cannabis both in Europe and the USA. At the end of 2022, HHC has been identified in seizures from the illegal market in 15 states: Denmark, Italy, Belgium, Sweden, Hungary, Estonia, France, Austria, Bulgaria, Spain, Lithuania, Czech Republic, Germany, Norway and Slovenia. To this concern, the European Monitoring Centre on Drug and Drug Addiction (EMCDDA) listed the molecule as New Psychoactive Substance (NPS) and posed it under strict control starting from November 2022 due to the abuse potential of this cannabinoid [12, 13]. Indeed, the synthetic cannabinoids class is the most representative NPS class, accounting for about 170 analogues detected in the illicit market of drugs so far [14, 15]. Considering the high number of synthetic cannabinoids related to intoxication, this class of substances represents an important public health harm to be monitored. Currently, potency, efficacy, and adverse effects are largely unknown, putting public health and safety at risk. Since the family of HHC-analogues is increasing, further studies are needed for a full pharmacological characterization of these chemicals and greater attention should be given to the toxicological effects and discard adverse effects.

In this scenario, we sought to review the literature on HHC from a pharmacological, toxicological and analytical point of view to fill the current gap on this emerging harm.

## 2 METHODS

A systematic literature search was performed in PubMed and Scopus databases and official international organizations’ websites. The keyword “hexahydrocannabinol”, was combined with “pharmacology”, “cannabimimetic activity”, “cannabinoid agonist”, “cannabinoid tetrad”, “phytocannabinoid”, “toxicity”, “toxicology”, “side effects”, “analysis”, and “detection”. A total of 172 scientific articles published until 2023 were initially screened for eligibility. Two scientists individually evaluated each entry from databases by reading only titles and abstracts mentioning relevant information. Further screening excluded studies according to the following criteria: articles written in English, Italian, or French language, articles about the synthesis and the pharmaceutical chemistry and containing irrelevant data. All the duplicate sources were removed. A total of 46 publications have been finally included.

## 3 RESULTS AND DISCUSSION

### 3.1 Pharmacology

The HHC shows the typical cannabinoid effects both *in vivo* and *in vitro*. However, several pharmacological studies have demonstrated that HHC is less potent than its precursor in various animal species (*e.g*., monkeys, dogs, gerbils, mice and rats) [16-21]. In particular, the rhesus monkey test, considered the most suitable and relevant test for evaluating the psychotropic activity of cannabinoids, confirmed that HHC is less potent when compared to Δ^9^-THC. Moreover, the *in vitro* affinity toward the cannabinoid receptors is different for 9(S)-HHC and 9(R)-HHC, exerting different behavioral and pharmacological effects [17, 22, 23]. Cannabinoid-induced tetrad is a preclinical model commonly used to assess the affinity of a pharmacological compound on the type-1 cannabinoid (CB_1_) receptor in the central nervous system. Tetrad is characterized by hypolocomotion, hypothermia, catalepsy, and analgesia, four phenotypes that are induced by acute administration of CB1 agonists exemplified by the prototypic cannabinoid Δ^9^-THC [24]. In this concern, controversial results on tetrad tests were reported *in vivo* for 9(S)-HHC and 9(R)-HHC. In fact, a study showed that only 9(S)-isomer presented a typical cannabimimetic profile evaluated *in vivo* by the tetrad test [25].

As far as the analgesic properties of HHC, the literature is controversial. Behavioral and pharmacological evaluations demonstrated that, although both isomers have the psychopharmacological activity of the cannabinoids, only 9(R)-isomer showed analgesic activity equipotent with morphine and Δ^9^-THC in the mouse hot plate test [25]. Differently, another study revealed that both epimers showed no difference in analgesic activity [23]. The mechanism of the analgesic activity of HHC is not well-established, but could, at least partially, be explained by the allosteric modulation of μ and δ opioid receptors of neuronal membranes, as already proved for Δ^9^-THC [21, 26]. In fact, it has been demonstrated that HHC is able to reduce dose-dependently *in vitro* binding of [3H]dihydromorphine (μ-opioid receptor) [19]. These preclinical results suggested the considerable structural specificity for the cannabimimetic activity suggesting fundamental differences in the mechanism of action of HHC and Δ^9^-THC.

Moreover, the abuse liability and dependence potential of HHC are still in their infancy. Although HHC is less potent than Δ^9^-THC, we cannot rule out drug dependence and drug abuse liability since HHC is structurally related to Δ^9^-THC and displays CB1-mediated tetrad effects in mice. Moreover, it is possible to postulate that 9(S)-HHC and 9(R)-HHC may have different abuse potentials since both isomers displayed different *in vitro* and *in vivo* affinity toward the cannabinoid receptors. 9(S)-isomer seems to be the more psychoactive due to the typical cannabimimetic profile evaluated *in vivo* by the tetrad test [25]. Therefore, a great deal of additional work is needed to establish acute and lasting behavioral, psychological, and neurological effects in animal models (conditioned place preference and dopamine *in vivo* microdialysis). The latter experiments may represent an interesting topic for future research.

The pharmacodynamics of HHC has been poorly investigated, and most of the evidence referred to HHC-analogues that have been synthesized to achieve molecules with a better profile of side effects compared to that of Δ^9^-THC. Indeed, the principal psychoactive constituent of cannabis, despite its beneficial uses for managing several health disturbances (*e.g*., chronic pain, chemotherapy-induced nausea and vomiting, and multiple sclerosis) [27, 28], shows serious side effects due to its ability to modulate the two Gi/o-protein coupled cannabinoid type 1 (CB_1_) and type 2 (CB_2_) receptors that are widely distributed in the body [29-31]. The central side effects of cannabinoids are mainly CB_1_ receptor-mediated, modulating numerous neurobiological processes in the central nervous system [29, 30]. Since CB_1_ receptors interact with a broad group of structurally diverse ligands [32], it has been postulated that the stereoselectivity of HHC might increase the affinity of the novel agonists toward desired subsets of signal transduction pathways and reduce the affinity toward signaling pathways that mediate undesirable side effects. Since the 1990s, Mechoulam and Makriyannis, with their colleagues, have designed and synthesized key pharmacological endocannabinoid molecules that are HHC-analogues [23, 33]. Therefore, there has been considerable effort to understand their likely therapeutic involvement in several diseases by targeting the cannabinoid receptors by synthesizing new compounds that selectively interact with cannabinoid receptors. Taking into consideration this purpose, numerous synthetic cannabinoid receptor agonists have been synthesized and characterized, modifying the HHC chemical scaffold and increasing target selectivity. Therefore, the tricyclic HHC ring modifications produced novel functionalized compounds and their putative cannabimimetic activity have been tested in preclinical studies measuring the cannabinoid tetrad [23, 34]. Interestingly, consistent disparities have been observed between the potencies and efficacies of structurally different cannabinoid agonists, confirming that the cannabinoid receptor site has a stereochemical requirement. Among the others, AM11245 and AM841, two HHC-analogues, exhibited high binding affinities for CB_1_/ CB_2_ receptors and cannabimimetic properties *in vivo* and *in vitro* tests with their Ki values in the picomolar range; nevertheless, the former showed more affinity for peripheral cannabinoid receptors whilst the latter exhibited its agonist profile both centrally and peripherally when tested *in vivo* [34]. In particular, *in vitro* test (the inhibition of forskolin-stimulated cAMP accumulation) demonstrated that AM841 acts as a full agonist of CB_1_ receptors whilst Δ^9^-THC behaves as a partial agonist [35]. Future binding studies and pharmacodynamics *in vivo* evaluations are needed to better establish the mechanism and potential value of the pharmacological and toxicological effects of HHC and HHC-analogues, which might modulate others than the CB_1_/CB_2_ and opioid receptors, like the transient receptor potential (TRP) channels of both the vanilloid type-3 (TRPV3) and the ankyrin type-1 (TRPA1).

### 3.2 Toxicology

**Fig. (2):**
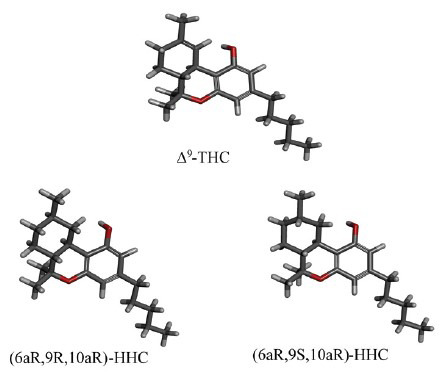
The tridimensional configuration of HHC epimers compared to the tridimensional configuration of Δ^9^-THC.

Scarce data on HHC toxicity are available due to the paucity of specific *in vitro* and *in vivo* studies. In addition, most of the studies were carried out before the cannabinoid system was discovered. However, the lower potency of HHC compared to Δ^9^-THC was first described in 1940 through an *in vitro* bioassay [5, 36]. Interestingly, the 9(S)- and 9(R)-HHC epimers showed different “cannabis” effects in a squirrel monkey model intravenously and intraperitoneally administered with different cannabinoids. Independently of the administration route, the 9(R)-HHC was efficacious in the decrease of response rate at lower doses (0.1-0.002 mg/kg dose) than Δ^9^-THC and CBD. Notably, the 9S-epimer was completely inactive even at high doses (3 mg/kg), suggesting that the behavioral effect may depend on the tridimensional configuration of 9(R)-HHC, which is more similar to Δ^9^-THC (Fig. **2**). To this concern, the 9(R)-HHC is considered the most toxic epimer [37]. Several HHC analogs have been synthesized and studied as candidate drugs but their toxic potential excluded further development, such as the adamantil-substituted analogue AM4045 [9]. However, these molecules allowed investigating of the cannabinoid receptors’ functions and the structure-activity relationship of cannabinoids. Moreover, the CB_1_-receptor agonist activity may suggest an addictive potential [38].

The HHC *in vivo* effects on humans have not been described yet, since real intoxication cases involving the HHC were not disclosed, probably due to the lack of proper analytical assays able to detect it in biological matrices. Indeed, the unintentional HHC exposure to “light” cannabis products is supposed to be due to the recent characterization of the molecule in several commercial products [39].

### 3.3 Analytical Detection

To date, different analytical methods have been developed to determine the diastereomers of HHC and other different cannabinoids in non-biological matrices, whereas no assay has been reported in biological matrices (Table **1**). Since 9(S)-HHC and 9(R)-HHC are diastereomers, the separation can also be carried out by achiral chromatographic methods. Most published methods used achiral chromatographic methods coupled to mass spectrometry (MS) as well as high-resolution mass spectrometry (HRMS). Only Collins and colleagues developed an analytical method using supercritical fluid chromatography for the separation of chiral molecules [40]. In 2022, Karin and colleagues developed a high-performance liquid chromatography-tandem mass spectrometry (HPLC-MS/MS) method for the separation and quantitation of emerging Δ^9^-THC isomers and analogues, including HHC, ∆^8^-THC, ∆^6a^,^10a^-THC, [41]. The method separated both THC isomers and/or isobars [41]. In the same year, Smith and colleagues used gas chromatography-mass spectrometry (GC-MS) and Direct Analysis in Real Time coupled with a time-of-flight mass spectrometer (DART TOF-MS) to investigate 26 different commercially available e-liquids for the presence of “hemp-derived” natural and unnatural cannabinoids [42]. A variety of cannabinoids, including HHC, ∆^8^-THC, ∆^9^-THC, ∆^6a^, and ∆^10a^-THC, were identified. Calibration was achieved using polyethylene glycol 600 in methanol, and a quality control containing cocaine, methamphetamine, and nefazodone was used to confirm masses fall within 5 mmu of the target along the m/z range. Mass spectra from the DART TOF-MS and GC-MS retention times were compared to available analytical reference material. If the reference material was not available, the ∆^9^-THC isomers and derivatives (Table **1**) were identified, comparing the spectra to those reported in NIST, swgdrug, and Cayman spectral libraries. The GC-MS method was successful in separating and identifying the “hemp-derived” ∆^9^-THC isomers and associated isobars unlike the DART TOF-MS method, which only succeeded in identifying the “hemp-derived” ∆^9^-THC isomers and derivatives [42]. HPLC and GC-MS methods to separate the two epimers from other cannabinoids, such as CBN, CBD, (-)-trans- Δ^8^-THC, (-)-trans-Δ^9^-THC, were applied to several natural and non natural HHC sources by Sams [43].

The recent availability of reference standards for both epimers allowed the complete chromatographical resolution and quantification of those compounds by GC-MS. To this concern, the more psychoactive isomer α-HHC eluted after β-HHC under the applied instrumental conditions [43]. Complete separation of the diastereomers of natural HHC was also obtained by reversed-phase chromatography on C18 bonded phases and with a reversed elution order compared to an analysis by GC-MS [43]. Stothard and colleagues provided further information to obtain the correct identification and differentiation of the products resulting from the hydrogenation of Δ^8^-and/or Δ^9^-THC. The correct identification and differentiation of the diastereomers were carried out by GC-MS and HPLC. 9(S)-HHC and 9(R)-HHC were chromatographically separated by GC and showed only minor differences by MS, so identity cannot be inferred without verified reference standards. Separation of the HHC diastereomers was also possible by HPLC using an isocratic method [44]. At the end of 2022, a further new GC-MS method was developed by Casati and colleagues for HHC diastereomers determination. The method was fully validated according to Food and Drug Administration (FDA) guidelines for drugs. Linearity was considered satisfactory if r^2^ ≥ 0.990 and the coefficient of variation (cv) ≤ 15%. Precision and accuracy were determined by calculating the coefficient of variation (cv%) and the bias (bias%) [45]. In addition, they reported an LC-MS method to separate 9(S)-HHC and 9(R)-HHC, using an isocratic elution, that showed an opposite elution sequence of the two diastereomers compared with GC, as also seen experimentally by Sams [43]. Finally, Collins and colleagues described the separation of HHC by supercritical fluid chromatography (SFC) analysis. SFC is used for the separation of chiral molecules and uses CO_2_ as the mobile phase as well as the same columns as standard HPLC systems. The crude oil, obtained from the reaction mixture from the conversion of CBD, was dissolved in hexane, purified over silica (0 to 5% ethyl acetate), concentrated in a vacuum and then distilled to afford a mixture of the HHC epimers as a yellow oil. This oil was further purified by a preparative method, and the diastereomers were separated and characterized by an analytical method [46].

## CONCLUSION

Recently, HHC, a synthetic analogue of the natural Δ^9^-THC, was listed as NPS by the EMCDDA due to the rapid spread in the illegal market. Although the scarcity of pharmacological studies, HHC cannabimimetic effects emerged both in *in vitro* and *in vivo* studies, which were carried out in the first half of the last century. The toxic effects on humans may only be deduced since intoxication-related cases have not already been reported. Future binding studies and pharmacological *in vivo* evaluations are needed to better establish the mechanism and potential value of the pharmacological and toxicological effects of HHC and HHC-analogues. Similarly, to other NPS, HHC represents a cheaper alternative to the controlled Δ^9^-THC, since kitchen laboratories may easily produce it from natural sources. Monitoring this emerging cannabinoid analogue is a crucial challenge for toxicological and forensic purposes. To this concern, it is essential to further investigate the HHC toxicity to inform not only the scientific community but also to support health providers in the identification of related intoxications.

## Figures and Tables

**Table 1:** Analytical methods for diastereomers of HHC and other related cannabinoids determination in non-biological matrices.

**Sample**	**Analytes**	**Sample ** **Preparation**	**Instrument**	**Ionization ** **Source**	**Column**	**Mobile Phase**	**Run Time (min)**	**References**
e-cigarette liquids	HHC, ∆^8^-THC, ∆^6a^, ^10a^-THC, THC-P, THC-O	Dilute and shoot	HPLC-MS/MS	ESI+	Phenomenex Kinetex^®^ 2.6 μm C18 (100Å 150 x 3 mm)	Isocratic H_2_O:ACN (25:75 *v/v*) + 1 mg/mL ammonium formate + 1% formic acid	-	[41]
e-cigarette liquids	HHC, ∆^8^-THC, ∆^9^-THC, ∆^6a^,^10a^-THC, THC-O, THC-P, CBD-di-O	Dilute and shoot	GC-MS	EI	HP-5MS column (30 m, 0.25 mm id, 0.25 μm)	Helium	26.33	[42]
			DART TOF-MS	ESI+	-	Helium	-	
Pure standards	9(S)-HHC, 9(R)-HHC, (-)-trans-Δ^8^-THC, (-)-trans-Δ^9^-THC, 9α-OH-HHC, 9β-OH-HHC, ∆^6a^,^10a^-THC, CBN, CBD	Dilute and shoot	GC-MS	EI	30 m capillary 35% diphenyl/65% dimethylpolysi-loxane bonded-phase)	Helium	-	[43]
			HPLC-PDA	-	150 mm x 4.6 mm 2.7 mm particles (Raptor ARC-18, Cat # 9314A65)	5 mM ammonium formate + 0.1% formic acid (25%) + acetonitrile + 0.1% formic acid (75%)	-	
Pure standards	9(S)-HHC, 9(R)-HHC	-	GC-MS	EI	Restek Rtx-5 MS column	Helium	25-minute	[44]
		-	HPLC-UV	-	Gemini-C18 250 x 4.6 mm, 5 μm	20:80:0.1 Water/Methanol/ acetic acid	-	
Crude extract	9(S)-HHC, 9(R)-HHC	Preparative SFC	SFC	-	Column 4.6 x 100 mm Chiralpak AD-H from Chiral Technologies (West Chester, PA)	Isocratic elution CO_2_, Co-solvent Isopropanol	-	[46]
Hemp derived resin	9(S)-HHC, 9(R)-HHC	Extraction with hexane and derivatization with MSTFA	GC-MS	EI	Agilent capillary column CP sil 8 CB (15 m · 0.25 mm i.d., 0.25 µm film thickness)	Helium	13	[45]
		-	LC-QTrap	-	Acquity fluoro-phenyl column (130Å, 1.7 µm, 2.1x 100 mm) (waters)	Isocratic elution 0.1% formic acid in H_2_O: 0.1% formic acid in CAN (35:65 *v/v*)	-	

**Abbreviations:** ACN, acetonitrile; CBD, cannabidiol; CBD-di-O, cannabidiol-di-acetate; CBN, cannabinol; EI, Electronic Impact; ESI, Electrospray Injection; HHC, Hexahydrocannabinol; GC, gas chromatography, HPLC, High-Pressure Liquid Chromatography; MS, mass spectrometry; PDA, photodiode array; QTrap, quadrupole tandem ion trap; SFC, Supercritical Fluid Chromatography; THC-O, tetrahydrocannabinol acetate; THCP, Tetrahydrocannabiphorol; UV, Ultra Violet.

## LIST OF ABBREVIATIONS

FDA: Food and Drug Administration
GC-MS: Gas Chromatography-mass Spectrometry
HHC: Hexahydrocannabinol
HRMS: High-resolution Mass Spectrometry
MS: Mass Spectrometry
NPS: New Psychoactive Substance
SFC: Supercritical Fluid Chromatography
